# Does Obesity Increase the Risk of Dementia: A Literature Review

**DOI:** 10.7759/cureus.2660

**Published:** 2018-05-21

**Authors:** Ibrar Anjum, Muniba Fayyaz, Abdullah Wajid, Wafa Sohail, Asad Ali

**Affiliations:** 1 Internal Medicine, The University of Texas MD Anderson Cancer Center, Houston, USA; 2 Department of Internal Medicine, Fatima Memorial Hospital, Lahore, Pakistan, Lahore, PAK; 3 FMH College of Medicine & Dentistry, FMH College of Medicine & Dentistry, Lahore, Pakistan; 4 Dow Medical College, Dow University of Health Sciences (DUHS), Karachi, Pakistan; 5 Medicine, CMH Lahore Medical College and Institute of Dentistry, Lahore, PAK

**Keywords:** obesity and dementia, adiposity and dementia, body mass index and dementia, obesity linkage with alzheimer's disease

## Abstract

Obesity and dementia are both associated with an increased risk of Alzheimer's disease (AD), and underlying neurodegenerative changes. Review articles provide evidential support that obesity and dementia result in an early old-age memory crisis. Obesity triggering vascular dementia decreases not only blood supply to the brain, but also increases fat cells that damage the brain white matter leading to loss of cognitive and intellectual behaviour. Adipocyte-secreted proteins and inflammatory cytokines explain the association between obesity and increased risk of dementia. Late-life elevated body mass index (BMI) confers a lower risk of having dementia. The hormone leptin explained the mechanism for the reverse association. Future studies need to reveal the linkage between adiposity and excess risk of dementia and AD.

## Introduction and background

Obesity and dementia are two significant concerns in the modern day medical science as both are prevailing at an alarming rate. Obesity a term, which is misunderstood by many, is defined as a state in which the individual contains too much body fat. An individual who consumes calories more than he or she burns can achieve this state which leads to various complications of cardiovascular disease, cognitive disabilities, sleep disorders, and joint complications [1-3]. Body mass index (BMI) is used to calculate body mass of an individual. The interpretations give result calculated by height and weight and scale them accordingly. Although there are many interpretations, variations and limitations in the BMI assessment, it provides one of the best measurements and depth of fatness in adults [4]. The belief of dementia being a single memory-related disorder of Alzheimer’s disease (AD) has vastly overgrown. Today's understanding of dementia is a comprehensive loss of memory with reduced cognitive and intellectual performance due to damaged brain cells. The magnitude of this impairment vigorously hinders the activity of emotions, speech and mental alertness allowing to abnormally elevated protein levels of tau proteins and β-amyloid within the brain [5-7]. Although there may be ways to increase the cognitive and behavioural functioning of the individual with provided medications, dementia continues to remain the topic of discussion and ongoing research in the disorders of modern-day old age [8]. An individual carries approximately 5 liters or 1.5 gallons of blood. Increased body fat content would advocate for a decreased supply of blood to the brain, promoting to vascular dementia. Loss of vascular supply would result in ischemia of brain, causing an increased possibility of memory loss, therefore, long-term dementia [9-10]. Also, obese individuals tend to have higher levels of adipokines or fat cells released by cytokines. These cells have been linked to decreasing white matter in the brain, which is responsible for nerve connections throughout the brain. Decreased neuronal functioning and decreased vascular supply to brain obsolete to brain atrophy and consequently loss of normal brain functioning [11-12].

## Review

The current existing literature on BMI and AD is conflicting and combining the results of many studies demonstrated a significant amount of contradicting information. A meta-analysis done on 16 articles reporting on 15 prospective studies showed that underweight, overweight and obesity in mid-life is associated with an increased risk of dementia when compared to a having normal body mass index [13]. The chances were highest for obesity, and the findings were similar for AD than any other form of dementia since the AD is the most prevalent form of dementia. Having an elevated BMI in midlife significantly increases the risk of dementia possibly due to increased inflammation, higher cytokine and hormone levels produced by adipose tissues [14]. Having an increased BMI can also be associated with a vast number of morbidities such as insulin resistance leading to diabetes, high cholesterol, hypertension and cardiovascular disease [15]. The vascular effects may also have a role in the development of a rapidly growing disease of late life, Alzheimer's disease. The factors as mentioned above and the higher BMI also related to the alterations in brain structure, white matter changes, blood-brain barrier disturbances and the age-related regulatory changes in protein, carbohydrate and lipid metabolism may influence dementia pathology. Furthermore, increased BMI and overweight individuals increase central adiposity and waist circumference. These changes are associated with white matter changes, disturbances of blood-brain barrier integrity and brain atrophy. Endocrine function alteration mediated by hormones and adipokines due to increased BMI could be another clue to underlying dementia later in life [16].

Low-dose organochlorine pesticides (OCPs) exposure is another risk factor for mid-life obesity and dementia. OCPs are lipophilic chemicals with half-lives of several years, and their exposure is increasing among the general population. OCPs are stored in adipose tissue, released and metabolize slowly over the years. Their serum concentration is the same as the concentration in the brain. Hence it can be stated that any condition that causes the release of OCPs from adipose tissue can increase the risk of dementia. Some of these conditions can include dysfunctional adipocytes causing uncontrolled lipolysis and weight loss. Since obesity is the most common cause of dysfunctional adipocytes, midlife obesity can increase dementia risk significantly [17].

Some other mechanisms that underlie the connections between obesity and the risk of cognitive impairment include insulin resistance, gut-brain axis, systemic mediators and central inflammation processes. It has recently been hypothesized that the gut microbiota could be part of a mechanism between the consumption of high fat and other unbalanced diets and impaired cognition known as 'gut-brain axis’. In metabolic disease, the inflammatory system becomes activated which can cause inflammation to spread from the peripheral system to the brain disrupting synaptic plasticity leading to neurodegeneration and eventually brain atrophy. A common trigger for this inflammatory activation can be found in the gut microbiota composition and therefore consuming a diet constituting high fats and sugars influences the microbiota composition, which may eventually lead to an imbalanced microbial population in the gut [18].

The chronic overconsumption of foods rich in carbohydrates and saturated lipids in obesity can affect insulin secretion and has a significant impact on cerebral glucose metabolism. The common intracellular mechanisms in type 2 Diabetes Mellitus and AD include aberrant redox regulation, oxidative stress, and active inflammatory processes resulting in impaired insulin secretion and signalling pathway. The methods to reduce the risk of complications associated with insulin resistance and diabetes also show benefits for reducing the risk of the AD, for example, regular physical activity and adherence to a fat- and carbohydrate-controlled diet [19]. Excessive insulin also invokes synchronous increases in levels of Abeta and inflammatory agents, effects that are exacerbated by age and obesity [20]. Another study is supporting the statement mentioned above on 12 male Wistar rats that were divided into two groups, one with a 50% fat diet and the other with a 10% fat diet and were assessed weekly for weight gain. This study demonstrated that increased weight gain in the rats led to insulin resistance and a possible further decrease in cognitive decline. Overall, it is well known that insulin is essential for the cognitive development of the brain and its deficiency or resistance in obesity can cause decrease insulin transfer from the peripheral nervous system to the brain hence leading to dementia [21].

Stress can also be a potential risk factor promoting abdominal obesity that can further increase grey and white matter atrophy contributing to impaired cognitive function [22]. Obesity can also induce endothelial dysfunction and cause cerebral hypoperfusion and enhances the production of β-amyloid that tends to undermine endothelial function further, creating a vicious cycle leading to pathogenic changes of AD. This endothelial dysfunction is due to a decrease synthesis and actions of nitric oxide (NO) derived from the endothelium and increasing the production of oxidative stress. Increased plasma levels of asymmetric dimethylarginine decrease the production of NO by inhibiting NO synthase activity which eventually leads to cerebral hypoperfusion and cognitive and neurodegenerative changes in AD [23]. Obesity is also known to be a substantial risk factor for hypertension which in turn is a risk factor for ischemic white matter lesions, silent infarcts, generalized atherosclerosis, and cardiovascular morbidity and mortality. Several studies reported that high blood pressure precedes Alzheimer's disease for decades, but blood pressure decreases years before dementia onset and is lower in individuals with Alzheimer's disease than in controls. High blood pressure is related to the neuropathological manifestations of Alzheimer's disease such as AD encephalopathy accelerating the AD process [24].

In contrast, to the statements mentioned above, obesity in middle age can also increase the risk of future dementia independently of comorbid conditions [25]. Although many studies are supporting the link between obesity and dementia, there have been many studies that discuss the contrary. The review presented above done on multiple epidemiological studies also illustrated that late-life elevated BMI confers a lower risk of having dementia. The possible mechanism accounting for the reverse association was explained by the hormone leptin which is mainly secreted by the adipose tissue. An increased level of leptin regulates synaptic plasticity in the hippocampus and amyloid B-processing, hence decreasing dementia. Furthermore, this article also discussed late-life low BMI or underweight had an increased risk of having dementia when compared with normal range or stable BMI. This was possibly due to dementia pathology and many other factors associated with a low BMI which included eating behaviour, appetite, food choice, apathy, loss of initiative and decreased olfactory function which also observed in early stages of dementia. In addition, a meta-analysis done in China and the United States discussed that in later life (>60 years) an increase in BMI confers a lower risk for dementia. This can be due to several factors which include differential survival, the neglect of confounders, or low BMI is a marker of an underlying dementia process in the elderly [26]. In contrast, this study also discussed that midlife BMI is linked with an increased risk of dementia later in a person's lifetime.

Another observation study was done on two million people that also demonstrated low BMI was associated with an increased risk of dementia like other studies done in the past [27]. This study, however, did not overcome the problems of reverse causation and confounding. Hence this hypothesis was further tested by another study that accounted for Mendelian randomization. Mendelian randomization study was done on a large international homogenous population to avoid reverse causation and confounding by using genetic variants in the human population. The genetic variants are determined at conception and remain constant throughout an individual's life hence they are not influenced by reverse confounding. The principal outcome of this study was that a lifelong low BMI due to genetic variation in BMI-related genes was not associated with a high risk of dementia in contrast to the latter observational study which failed to demonstrate causation and needs a further explanation [28]. Furthermore, a prospective study that took an age (mean age: 70.8) into consideration shows that overweight and obesity with increased BMI in elderly is not associated with an increased risk of dementia. It discussed that an increased BMI later in life should not be taken as a risk factor for dementia [29].

Finally, a collaborative study of over 1.3 million adults from Europe, the United States, and Asia illustrated an increased dementia risk when weight was measured >20 years before dementia diagnosis. However, this association was reversed when BMI was assessed <10 years before dementia diagnosis [18].

Two different processes explained this finding, one being a direct causal harmful association between BMI and dementia if there is an extended follow-up and second explained by reverse-causation of BMI being a protective factor once the follow-up is short, as BMI assessment near the onset of dementia was biased by preclinical dementia. The study analyzed that higher baseline BMI increased the risk of all-cause mortality before the age of 65 years but lower mortality risk after the age of 85 years. The study suggested that obese individuals are more likely to die due to complications of diabetes and cardiovascular diseases compared with their normal-weight counterparts and they have less likely to live long enough to develop dementia. In these findings, the differences in survival may have contributed to an underestimation of the strength of the association between BMI and dementia [30].

## Conclusions

We have briefly discussed the relationship between obesity and dementia. Many factors can associate them together, some of which include co-morbid conditions arising from obesity such as insulin resistance diabetes, hypertension and cardiovascular disease which can, in turn, have negative consequences on the brain. A high BMI increases the risk for dementia due to bioactive hormonal compounds that are secreted by adipose tissue. If we understand the relationship among obesity and dementia further in details, it can help us take the necessary therapeutic measurements that can prevent cognitive decline in the future. Although many studies are supporting the relationship between the two, many studies are discussing the opposite. Overall, further research is still needed to support and validate our article.
